# Frequent and Recent Human Acquisition of Simian Foamy Viruses Through Apes' Bites in Central Africa

**DOI:** 10.1371/journal.ppat.1002306

**Published:** 2011-10-27

**Authors:** Edouard Betsem, Réjane Rua, Patricia Tortevoye, Alain Froment, Antoine Gessain

**Affiliations:** 1 Unit of Epidemiology and Pathophysiology of Oncogenic Viruses, Department of Virology, Institut Pasteur, Paris, France; 2 Centre National de la Recherche Scientifique (CNRS), URA 3015, Paris, France; 3 Faculty of Medicine and Biomedical Sciences, University of Yaounde I, Yaounde, Cameroun; 4 Institute of Research for Development, Musée de l'Homme, Paris, France; SAIC-Frederick, United States of America

## Abstract

Human infection by simian foamy viruses (SFV) can be acquired by persons occupationally exposed to non-human primates (NHP) or in natural settings. This study aimed at getting better knowledge on SFV transmission dynamics, risk factors for such a zoonotic infection and, searching for intra-familial dissemination and the level of peripheral blood (pro)viral loads in infected individuals. We studied 1,321 people from the general adult population (mean age 49 yrs, 640 women and 681 men) and 198 individuals, mostly men, all of whom had encountered a NHP with a resulting bite or scratch. All of these, either Pygmies (436) or Bantus (1085) live in villages in South Cameroon. A specific SFV Western blot was used and two nested PCRs (*polymerase*, and LTR) were done on all the positive/borderline samples by serology. In the general population, 2/1,321 (0.2%) persons were found to be infected. In the second group, 37/198 (18.6%) persons were SFV positive. They were mostly infected by apes (37/39) FV (mainly gorilla). Infection by monkey FV was less frequent (2/39). The viral origin of the amplified sequences matched with the history reported by the hunters, most of which (83%) are aged 20 to 40 years and acquired the infection during the last twenty years. The (pro)viral load in 33 individuals infected by a gorilla FV was quite low (<1 to 145 copies per 10^5^ cells) in the peripheral blood leucocytes. Of the 30 wives and 12 children from families of FV infected persons, only one woman was seropositive in WB without subsequent viral DNA amplification. We demonstrate a high level of recent transmission of SFVs to humans in natural settings specifically following severe gorilla bites during hunting activities. The virus was found to persist over several years, with low SFV loads in infected persons. Secondary transmission remains an open question.

## Author Summary

Most of the viral pathogens that have emerged in humans during the last decades have a zoonotic origin. After the initial interspecies transmission, these viruses have followed different evolutionary routes and have spread among humans through distinct mechanisms. The understanding of the initial steps of the emergence of several viruses and associated diseases often remains quite poor. Human infection by simian foamy viruses (SFV) can be acquired by persons occupationally exposed to non-human primates (NHP) or in natural settings. Epidemiological and microbiological studies in specific high-risk populations are necessary to gain new insights into the early events of the emergence process, and the potential to spread or cause disease among humans. The present study found that hunting is still a very common and risky activity for SFV infection in forest areas of South Cameroon. Indeed, recent interspecies transmission of SFVs to young adults is still very frequent, as 1 person out of 5 among the hunters who have reported a bite or scratch by a non-human primate and 2 persons out of a thousand in the general population are persistently infected by a SFV, mostly from an ape. Secondary transmission to other family members and presence of a disease in infected persons are still open questions that are being investigated.

## Introduction

Most of the viral pathogens that have emerged in humans during the last decades have originated from various animals, either domestic or living in the wild [1], [2], [3], [4]. After the initial interspecies transmission, these viruses have followed different evolutionary routes and spread into the human population through various distinct mechanisms. Such mechanisms have been well studied, often well understood, thus allowing a certain level of risk control, and a decrease of inter-human dissemination [3], [4], [5]. In contrast, the understanding of the initial steps of the emergence of several viruses and associated diseases often remains quite poor. Epidemiological and microbiological studies in specific high-risk groups and populations are thus necessary to gain new insights into the early events of the emergence process.

Nonhuman primates (NHPs) are hosts for several pathogens potentially transmissible to humans. Indeed, people in contact with NHPs are at risk for infection with viruses such as Simian T Lymphotropic Viruses [6], [7], [8], [9], [10], [11], [12] or Simian Immunodeficiency Viruses through interspecies transmission [13], [14], [15], [16], [17]. Simian foamy viruses are exogenous complex retroviruses of the *Spumaretrovirinae* subfamily [18], [19]. They are highly prevalent in several animal species, in which they cause persistent infection [20], [21], [22], [23]. Switzer et al., suggested that foamy viruses have co-speciated with Old World NHPs for at least 30 million years [24]. Such a long-term co-evolution may explain their apparent lack of pathogenicity observed *in vivo*, and the persistence of the infection. Indeed, SFVs are considered to be non pathogenic in naturally or experimentally infected animals, even though disease association has not been systematically evaluated in any NHPs species. This strongly contrasts with the *in vitro* cytopathic effect seen in infected cell cultures, with a characteristic foamy appearance of vacuolized cells [25].

SFV seroprevalence in captive adult NHP populations can reach 75–100% [26], [27], [28], [29], [30]. The situation seems very similar in semi-free ranging colonies [31], [32] and in wild troops [33], [34], [35]. Transmission of SFV among NHPs occurs via infected body fluids, mainly through biting, but also with grooming and possibly to a lesser extent, sexual contacts [32], [34]. SFV appears to be present at high concentration in the saliva of infected animals [36], [37], [38] and viral replication has been shown to occur in a superficial cell niche of the oral mucosa in macaques [38].

The first FV to be isolated in humans was reported by Achong in 1971 [39]. This virus was identified in a cell culture from a Kenyan patient suffering from a nasopharyngeal carcinoma. Further phylogenetic analysis indicated that this virus was from an east African Chimpanzee subspecies and the virus was now renamed “the prototype HFV” [40]. However, the first clear evidence of SFV in humans was demonstrated in 1995 by Schweizer et al., who found antibodies directed against SFV antigens and the presence of FV DNA in the peripheral blood of 3 persons among 41 laboratory and animal house personnel [27].

These initial studies were followed by series of others, mainly by a CDC team led by Dr W. Heneine and W. Switzer who published a series of clear demonstrations of the presence of SFV infection in cohorts of workers occupationally exposed to NHP, including animal caretakers, research scientists, and veterinarians [41], [42], [43], [44]. In most cases, the supposed infecting contacts were bites, from chimpanzees and African monkeys and, to a lesser extent, puncture wounds. In rare cases, no evident risk factors were identified, suggesting that other cutaneomucous contacts can also lead to such zoonotic infection [44], [45]. The next step was to search for such zoonotic infection in a more natural setting. Wolfe el al. pioneered the work by investigating the presence of SFV in villagers of South Cameroon reporting direct contacts with blood and/or body fluid from wild NHPs. This study demonstrated the presence of antibodies directed against SFV in 1% of the 1099 tested individuals and the presence of SFV sequences in the blood of 3 persons [46]. Our team has developed and extended such results in South Cameroon, demonstrating the presence of persistent SFV infection in a series of 13 individuals all, except one, being men bitten during hunting activities in the forest, by an ape or a monkey [47]. Studies in South-east Asia showed transmission of macaque SFVs in a series of 10 people including zoo workers, owner of NHP pets, bush meat-hunters and temple workers [33], [48]. Furthermore, mathematical modeling showed that in Bali, about six of every 1000 visitors to monkey temples will be infected by SFV [49].

In an area of high NHP diversity and ongoing ecologic and socio-demographic changes, the goals of the present study were to gain new insights into the risk factors associated with the presence of SFV infection in human populations neighboring a nature reserve rich in game. In this area, hunting and butchering for subsistence are still very active. A second goal was to characterize SFV strains and viral loads in peripheral blood of infected individuals and finally, we searched for any intra-familial dissemination of SFV from the originally infected index cases.

## Materials and Methods

### Clearance and ethics

The study received administrative and ethical clearance in Cameroon from the research division of the Ministry of Public Health (reference D30-295/AR/MINSANTE/SG/DROS/CRC/CEA1) and from the National Comity of Ethics (reference 034//CNE/MP/06), and in France, from the “Comité de Protection des Personnes” (reference 2011/01NICB) and the “Commission Nationale de l'Informatique et des Libertés” (reference EGY/FLR/AR111711). Prior to field sampling, community and individual written informed consent was provided by participants after detailed information and explanations of the study were provided. Written consent for children underage was obtained from their parents or recognized guardians.

### The population

**Figure 1:**
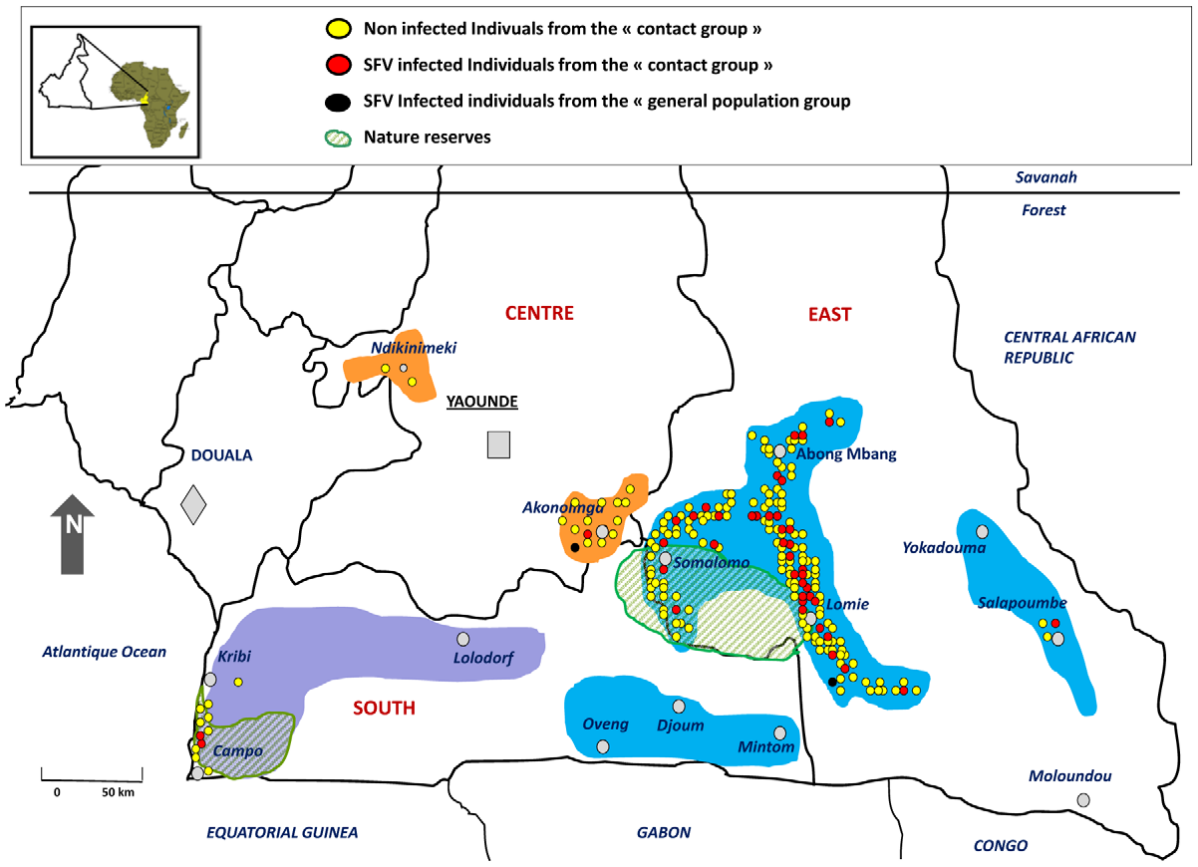
Geographic distribution of the studied population. Samples were collected systematically in the coloured areas without specific focus on a particular site. Native inhabitants of these areas include a great variety of ethnicities among which are the Banen, Yebekolo and Soo in the Centre (orange areas), the Bakola Pygmies, Mvae and Ngumba in purple colored area and finally the Baka Pygmies, the Bulu, Fang Badjoue and Zime tribes located in the blue coloured areas. The 198 individuals from the “contact group” are indicated by red (SFV-infected) and yellow (SFV non-infected) dots. The 2 SFV infected individuals from the “general population” are represented as black dots.

This study was carried out in rural areas located in south and east Cameroon (figure 1) in a rainforest region home to a variety of non human primate (NHP) species. The human populations in these areas include numerous Bantu tribes including. Pygmies in this work are from the Baka and the Bakola tribes [50]. A large part of this study was focused on areas and villages surrounding and within the Dja and Campo Maan nature reserves (figure 1). A systematic approach for the enrolment of adults was carried out in the populations (Pygmies and Bantus) in all reachable villages and settlements, scattered alongside roads and tracks across the forest. A standardized questionnaire was used to collect personal epidemiological data and two study population groups were defined. A large group designated “general population”, included all consenting subjects who had been living in the study areas for several years and been exposed to NHPs. A second group, smaller in size, designated the “contact group”, made up of all those individuals who had reported an encounter with a NHP during their lifetime, and which has resulted in physical injury by a scratch, a bite or both, from the animal in question. The classification into the two groups was made on the basis of a simple questionnaire, and an explicit declaration of an injury related to contact with a NHP, no matter what the circumstances. Collected data included the name, age, sex, location, ethnicity and family links, as well as specific questions about date of contacts with NHPs, the location, description of circumstances, the type and site of body lesions if any and the presence of after-effects.

A 5 to 10 ml whole blood sample was collected in EDTA K2 vacuum tubes, from all consenting individuals meeting the inclusion criteria. Plasma and buffy-coat were obtained 48 to 72 hours after sampling and kept frozen at −80°C.

A simple clinical examination was performed when requested by participants in the study. Treatment for common local ailments was given if available. A transfer to an appropriate medical facility was advised for severely ill individuals encountered on site.

### Serologic tests

All available plasma was screened with an experimental WB method, using a classical antigen produced in baby hamster kidney cells (BHK-21), infected by the prototype strain HFV [39] at a MOI (Multiplicity of infection) = 1. All samples were screened with a classical cell lysate antigen. Positive and indeterminate samples were tested anew with a concentrated purified antigen obtained from a culture supernatant, for clearer and more conclusive results. This antigen was produced from a cell lysate, filtrated through a 0.45 µm filter followed by 40 minutes of ultracentrifugation at 25000 rpm. The resulting concentrated pellet was suspended in 1× Laemmli buffer and kept frozen at −20°C. Antigenic 70 kDa and 74 kDa Gag proteins were separated by a 4 hour migration on polyacrylamide 10% bis tris gel (INVITROGEN, Aukland, New Zealand) with a direct 130 V filed. Antigens were transferred to a polyvinylidene fluoride (PVDF) membrane.

**Figure 2:**
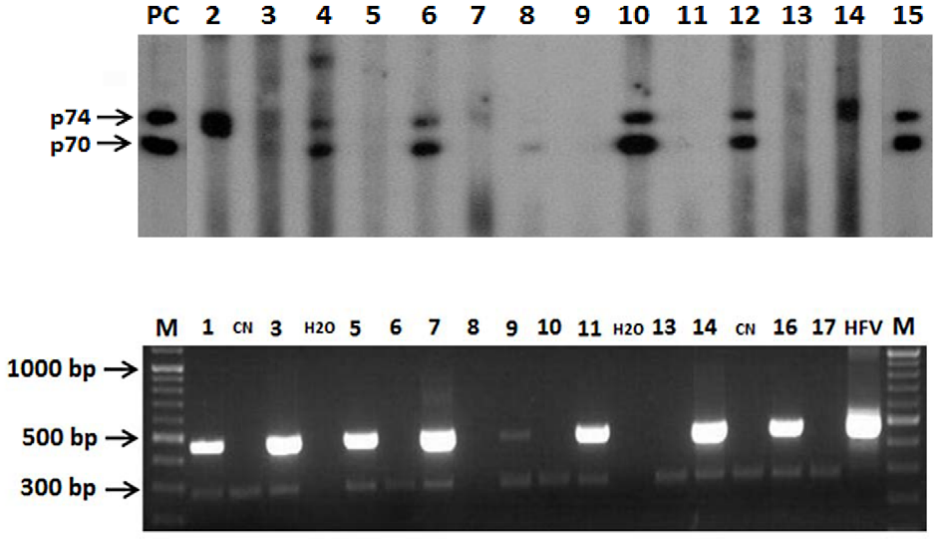
Serological and molecular results patterns for SFV detection. A) Western blot results using purified classical chimpanzee antigen sero-positive samples (lanes 3, 4, 6, 10, 12). Sero-indeterminate (lanes 2, 7, 8, 14). Sero-negative samples (lanes 5, 9, 11, 13). Positive SFV control serum from a gorilla-infected human (lane 15), and a macaque (lane PC). B) Nested PCR detection of 465 bp Integrase sequences of SFV M = molecular weight marker. CN = Negative Control. HFV = Human foamy virus, positive control, H2O = Water.

Positivity in serology was considered as the presence of the p70 and p74 Gag doublet (figure 2-A). Samples showing only one of the two Gag proteins were considered indeterminate and absence of doublet was considered a negative result.

### Molecular studies

High molecular weight genomic DNA was extracted from the buffy-coat of all individuals whose plasma was WB positive or indeterminate for the Gag doublet with a blood extraction kit (Qiagen, Gmbh, Hilden Germany) and for all subjects in the “contact group”, independently of their WB result. Quantified DNA (Biophotometer RS 232 C; Eppendorf, Hamburg, Germany) was amplified (Mastercycler, epGradient; Eppendorf) for a 229 bp fragment of the β-globin gene with primers PCO4 and GH2O as previously described [51]. Two nested PCRs were carried out for the specific detection of SFV DNA. Amplification of a 465 bp fragment on the *pol*-In (*polymerase* gene-Integrase), was done for 35 cycles (30″ denaturation at 95°C, 30″ annealing at 55°C, 1′ extension at 72°C and final 7′ extension step at 72°C) using highly generic primers (POL1outse, POL2outas,POL3inse, POL4inas) as previously described [52]. The second PCR was hemi-nested and amplified a fragment of the LTR for 35 cycles (30″ denaturation at 95°C, 30″ annealing at 55°C, 30″ extension at 72°C and final 7′ extension step at 72°C), using generic primers (PBF1se, PBF2as and PBF3se) [53]. “Classical” criteria for SFV infection were defined as 1) clear positivity to WB and 2) positive PCR for the *pol*-In and/or the LTR DNA fragments. The few individuals with a positive PCR and a negative or indeterminate WB were defined as “non classical”. Amplified DNA was purified with a gel extraction kit (Qiagen, Gmbh, Hilden Germany), and inserted into a 3.9 pCR 2.1 plasmid vector (Invitrogen) with the Rapid DNA ligation kit (ROCHE). Plasmids were cloned in chemically competent Escherischia coli (Invitrogen). Two to four different bacterial clones were selected for plasmid extraction and purification using the quick plasmid minipreps kit (Invitrogen). EcoRI digested fragments were sequenced using universal forward T7 and reverse M13 primers.

### Sequence analysis and phylogenetic studies

For every selected clone, both forward and reverse amplified nucleotide sequences were aligned using “Clustal X alignment” software included in the DAMBE version 4.5.68 (Xia, X., Xie, Z., 2001). Only one clone was considered when sequences were found identical. A consensus sequence was built when one or more nucleotides variations were found. A final consensus sequence was built for every sample from its different clone's consensus sequences. Final sequences were aligned and compared to different old world NHP prototype sequences. According to Akaike Information Criterion (AIC), different evolutionary models were tested using PAUP software version 4.0b10 (Sinauer associates, Inc. Publishers, Sunderland, Massachussets). Phylogeny was performed with the neighbour joining method and the best tree was selected after a bootstrap analysis of 1000 replicates.

### (Pro)viral loads

Quantitative PCR assays for DNA (qPCR) were performed using the Eppendorf realplex master gradient detection system. We used SYBR Green Quantitect (Qiagen) in a 20 µl volume reaction containing 10 µl of SYBR Green buffer, 150 nM of each primer and a 500 ng DNA sample. Five primer pairs were designed in a region of the Integrase in the *polymerase* gene, conserved among all our sequenced gorilla foamy virus strains. Primers (GF5qpcr-TAGACCTGAAGGAACCAAAATAATTCC, and GR5qpcr-TCCTTCCTCATATTAGGCCACC) gave the best sensitivity (1 to 10 copies per 500 ng). They were designed to detect a 144 pb nucleic acid region of the gorilla FV *polymerase* gene. The optimized qPCR conditions used were as follows: 95°C for 15 min, 40 cycles of: 95°C for 15 s, 60°C for 30 s and 72°C for 30 s. To standardize qCPR, a 465-pb region that included the PCR target sequence from one primary isolate was cloned into a PCR cloning vector, TOPO TA cloning kit (Invitrogen). Known amounts of the target gorilla foamy virus sequence (from 1 to 10^4^ copies) were added to 500 ng of human genomic DNA from MS5 cell line (fibroblastic cell line) to generate DNA standard curves. In addition to a standard curve, each PCR run included a buffer-only and foamy virus negative DNA controls. DNA derived from PBMC or buffy-coat was used at 500 ng (75×10^3^ cell equivalents). A cellular albumin qPCR was done on each sample to normalize with cellular DNA content (albF-AAACTCATGGGAGCTGCTGGTT, albR-GCTGTCATCTCTTGTGGGCTGT). Each DNA sample was tested at least in duplicate. We checked in every individual assay the specificity of the primers by using a melting curve.

### Statistics

Statistical analyses were performed on Stata software. A univariate analysis was performed for risk factors, by the double entry Fisher exact method with a significance of p<0.05. A multivariate analysis was performed to identify the most pertinent factors associated to SFV infection.

Viral loads were compared across groups using Student t test after logarithmic transformation. Categorical variables were compared across groups using Chi-square and Fisher exact tests where appropriate. Multivariable logistic regression was performed to identify factors independently associated with SFV infection.

### Accession numbers

Bak50/JN049028, Bad179/JN049029, Bad202/JN049030, Bad436/JN049031, Ako254/JN049032, Pyl149/JN049033, Bad316/JN049034, Bad327/JN049035, Bobak153/JN049036, Bobak237/JN049037, Lobak89/JN049038, Sabak36/JN049039, Ako394/JN049040, Bak33/JN049041, Bak40/JN049042, Bak55cl3/JN049043, Bak55cl2/JN049044, Lobak2/JN049045, Bak46/JN049046, Bak56/JN049047, Bak74/JN049048, Bak82/JN049049, Bak132/JN049050, Bak133/JN049051, Bak177/JN049052, Bak224/JN049053, Bak228/JN049054, Bak232/JN049055, Bak270/JN049056, Bad332/JN049057, Bad350/JN049058, Bad348/JN049059, Bad349/JN049060, Bad447/JN049061, Bad456/JN049062, Bad463/JN049063, Bad468/JN049064, Bad551/JN049065.

## Results

### The population

A total of 1,321 individuals were included in the “general population” group (table 1). There were 965 Bantus (males: 502, females: 463) and 356 Pygmies (males: 179, females: 177). Ages ranged from 5 to 90 years old in the Bantus and from 5 to 81 in the Pygmies. Individuals in the Bantu group were older (mean age of 51 years) than in the Pygmies (43 years), p<10^−4^.

**Table 1:** Global description of the general population group and the contact group, and overall serology and PCR results.

	Ethnicity	Sex	Age range	Mean age	n	Serology			PCR	
						Pos N(%)	Ind N(%)	Neg	Pos N(%)	Neg
General pop	Bantus	M	5–88	51	502	4(0.8)	44(8.7)	454	0	48
		F	6–90		463	8(1.7)	45(9.7)	410	1(0.2)	52
	Pygmies	M	5–80	43	179	10(5.6)	20(11.2)	149	1(0.5)	29
		F	6–81		177	4(2.2)	18(10.2)	155	0	22
**Total**					**1321**	**26(2)**	**127(9.6)**	**1168**	**2(0.2)**	**151**
Contact group	Bantus	M	6–87	42	115	19(16.5)	17(14.8)	79	19(16.5)	17
		F	28–75		5	0	1(20)	4	0	1
	Pygmies	M	24–75	50	76	34(44.7)	11(14.5)	33	22(29)	23
		F	24–35		2	0	0	2	ND	ND
**Total**					**198**	**53(26.7)**	**29(14.6)**	**116**	**41(20.7)**	**41**

Pop: population n: number of individuals in each group Pos: positive samples, Ind: Indeterminate samples, Neg: Negative samples PCR positive included positive samples for *pol*-In and/or LTR.

In the “contact group”, 198 persons, mostly males (males: 192, females: 7), reported direct contact with a NHP and a resulting wound (table 1). Pygmies in this group were older (24 to 75; mean age = 50 years) than Bantus (6 to 87; mean age = 42 years), p<10^−4^.

### Encountered NHP species in the contact group

**Figure 3:**
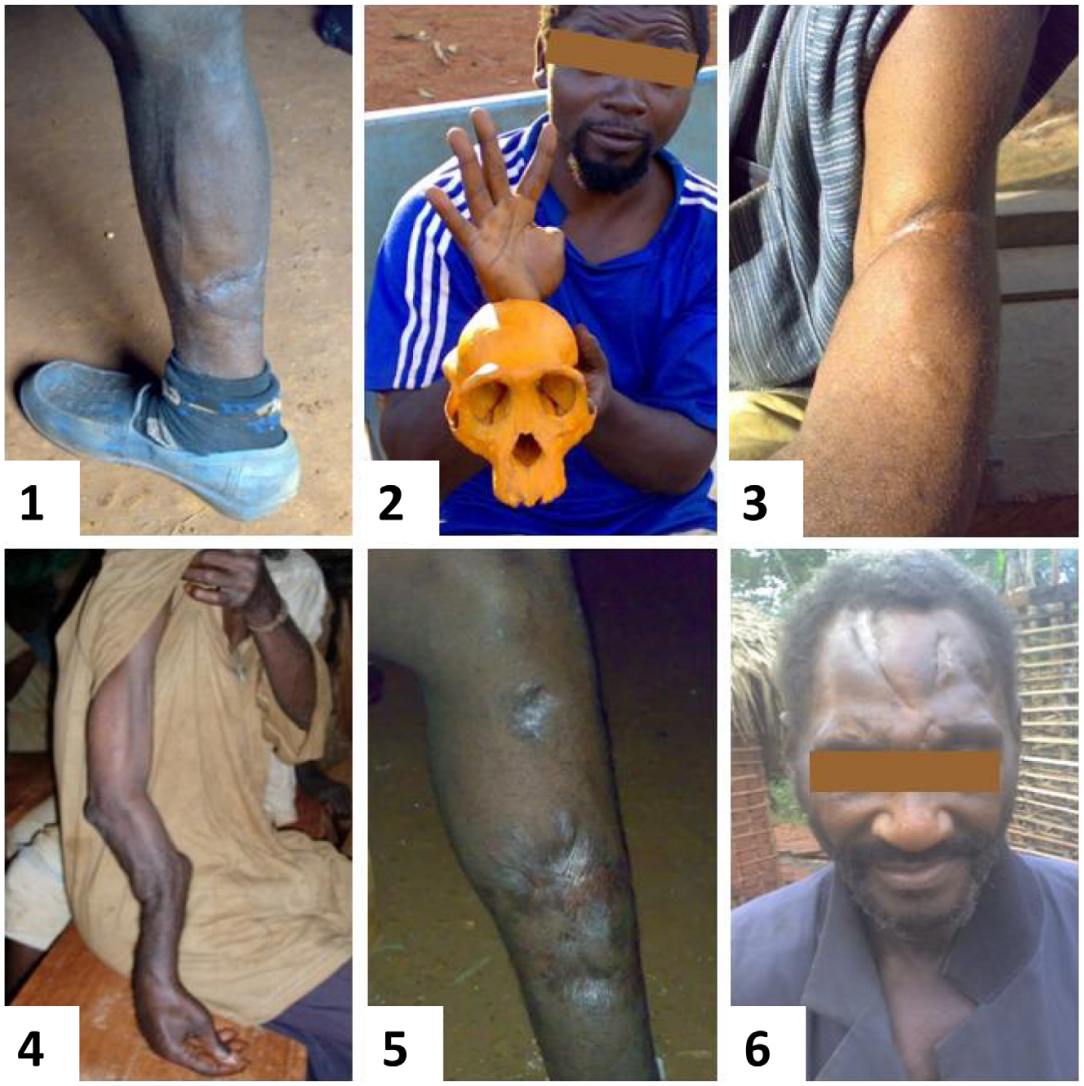
SFV infected individuals harboring scars and lesions caused by NHP bites. Scars from wounds by a small monkey in picture 1 (participant Bad50), chimpanzee in picture 2 (participant Bad327), gorilla in picture 3 (participant Bak56), picture 4 (participant Sabak36), picture 5 (Bad348), picture 6 (participant Bak132).

The different NHP species encountered by the hunters and reported in the “contact group” are native to the study area and are known to harbour specific SFVs [26], [34], [54]. Monkeys encounters were reported as often (103/198; 52%) as those with apes (95/198; 48%), (X^2^ = 0.65, p = 0.42). The most reported and recognized small monkeys were *Cercopithecus nictitans* (41/103; 39.8%), *C. cephus* (36/103; 34.9%) *and C. neglectus* (16/103; 15.5%). Baboons and mandrills (6/103, 5.8%) and other monkeys (4/103, 4%) were less frequently encountered. Among the apes, gorillas were three times more prevalent (74/95; 77.9%) than chimpanzees (21/95: 22.1%), p<10^−6^. Double contacts with NHPs were reported by only 3 individuals (2 Gorilla/Gorilla and 1 Chimpanzee/Monkey). Most of the individuals in the “contact group” still had a noticeable scar or permanent physical injury (figure 3).

Regarding ethnic distribution in the contact group, Bantus (81.5%; 84/103) reported more contacts with small monkeys than Pygmies (18.5%; 19/103) p<10^−4^. Chimpanzees and baboons were equally often encountered in both ethnic groups, while Pygmies (69%; 51/74) were more frequently in contact with gorillas than Bantus (31%: 23/74), p<10^−5^ (table 2).

**Table 2:** Species distribution of encountered NHP for the 198 individuals in the «contact group».

		Monkey species N(%)			Apes N(%)	
		*C. nictitans*	*C. cephus*	*Others*	Chimpanzee	Gorilla
Risk population	Bantus	36	29	19	12	23
	Pygmies	5	7	7	9	51
		**41(39.8)**	**36(34.9)**	**26(25.2)**	**21(22.1)**	**74(77.9)**

Description of the NHP species was done by the concerned individuals themselves and a record was made on the written questionnaire. Visuals were showed to help them precisely identify the animal. Others = *C. neglectus, Papio, Colobus, cercocebus, Mandrills.*

**Figure 4:**
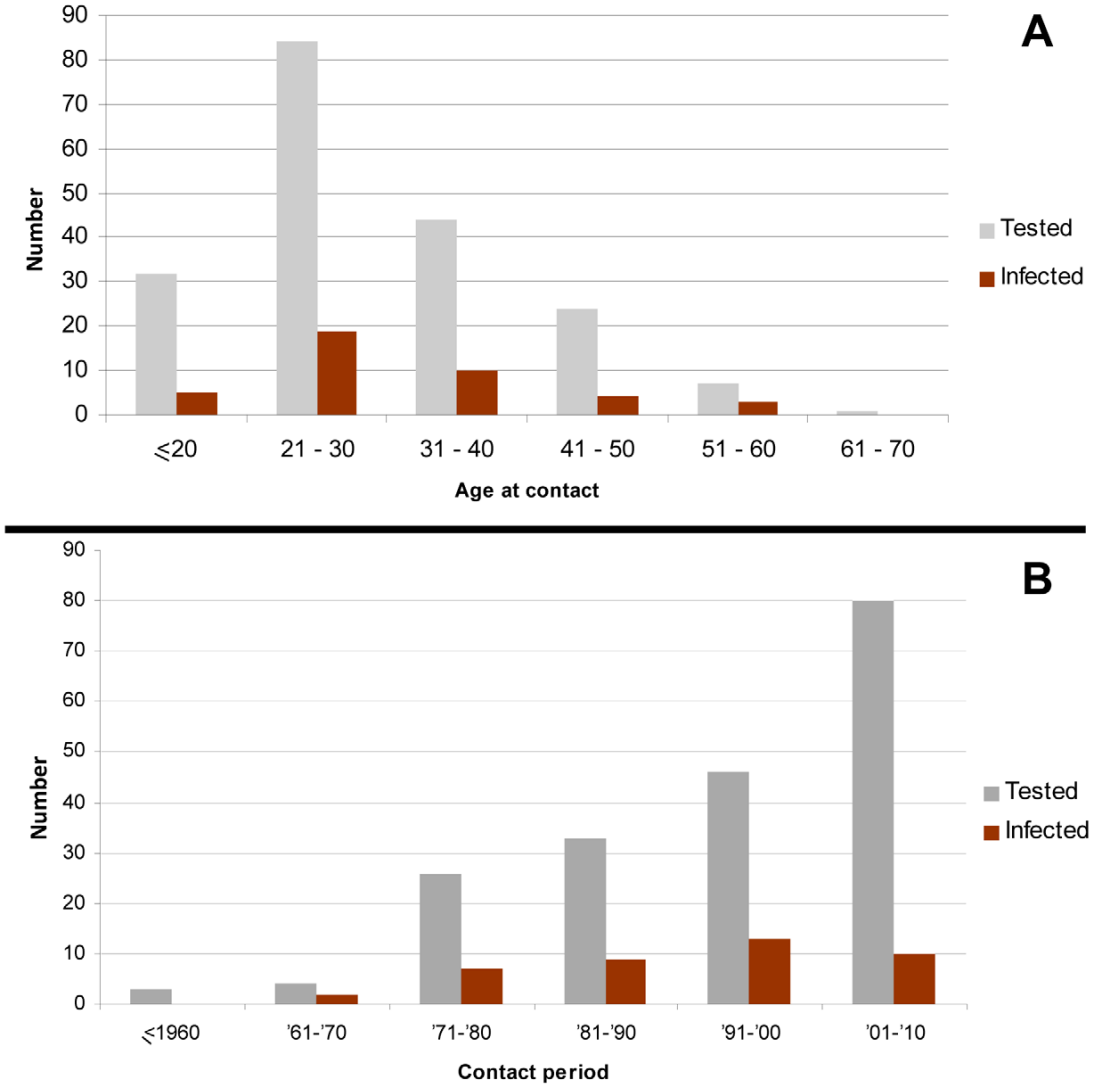
Distribution of tested and infected individuals from the “contact group”. A) According to the age at contact with NHP B) According to the time of the contact with NHP.

Overall, (83.3%) people aged up to 40 years are those most frequently involved in hunting and subsequent contacts with NHPs. Only (16.7%) were older, up to 70 years old, at the moment of contact (figure 4-A). Most of the encounters with a NHP (65.6%) had occurred within the last twenty years (range from 1991 to 2001) (figure 4-B).

### Serologic results

The WB results in the “general population” group, based on the chimpanzee antigen revealed 2% (26/1,321) clearly positive plasmas. Furthermore, 9.6% (127/1,321) of the tested samples showed an indeterminate Gag profile (figure 2-A). The remaining 1168 samples showed no Gag reactivity and were considered negative (table 1).

In the “contact group” WB analysis revealed 26.7% (53/198) of positive plasmas. Indeterminate serology was found in 14.6% (29/198) samples while the remaining 104 samples were clearly sero-negative (table 1).

### Molecular results

Buffy-coat DNA was available from all the individuals who exhibited a positive or indeterminate WB in the “general population” group (respectively 26 and 127) and in the “contact group” (respectively 53 and 29), and was amplifiable using β-globin specific primers.

Both *pol*-In and LTR PCRs were positive for two samples in the “general population” group (table 1, table 3). The 65 year-old Bantu woman (Ako254) and 68 year-old Baka Pygmy man (Bobak237) respectively reported frequent butchering activities of wild games, including NHPs and the man reported frequent hunting. None of them recalled known injury from a living NHP during their lifetime.

**Table 3:** Epidemiological and “classical” biological features of SFV infected humans in contact group and general population.

Groups	Ethnicity	Samp/sex	Age at: cont/samp	Samp periods	Wound loc/severity	Serology	PCR *pol*-In/LTR	Origin	(Pro)viral LoadΦ
Contact Group	Pygmies	Pyl149/M	45/60	2008	Hand/2	+	+/+	Cpz	ND
		Bobak153/M◊ ‡	53/59	2006/09	Hand/2	+	+/+	Gor	20
		Mebak65/MΔ	20/40	2006	Body/2	+	−/+	GorΨ	<1
		Lobak2/M◊ ‡	37/57	2006/09	Thigh/3	+	+/+	Gor	32
		Lobak89/M◊ ‡	20/50	2006/09	Arm/2	+	+/+	Gor	<1
		Sabak36/M	40/68	2006	Forearm/3	+	+/−	Gor	63
		Bak33/M◊ ‡	25/45	2008/09	Multiple/3	+	+/+	Gor	24
		Bak40/M	30/35	2008	Thigh/2	+	+/−	Gor	14
		Bak46/M◊ ‡	26/50	2008/08	Multiple/3	+	+/+	Gor	76
		Bak55/M‡	30/65	2008	Arm/2	+	+/+	Gor	145
		Bak56/M‡ ◊	40/65	2008/09	Hand/2	+	+/−	Gor	57
		Bak74/M‡ ◊	26/47	2008/09	Foot/2	+	+/+	Gor	117
		Bak82/M‡ ◊	46/50	2008/09	Leg/2	+	+/+	Gor	36
		Bak132/M◊	30/61	2009/10	Head/3	+	+/+	Gor	27
		Bak133/M‡ ◊	30/51	2009/10	Several/3	+	+/+	Gor	2
		Bak177/M‡ ◊	26/36	2009/10	Leg/3	+	+/+	Gor	122
		Bak188/MΔ	15/48	2009	Hand/1	+	−/+	CercoΨ	ND
		Bak224/M‡	19/38	2010	Several/3	+	+/+	Gor	28
		Bak228/M‡	29/70	2010	Foot/3	+	+/+	Gor	34
		Bak232/M‡	40/60	2010	Hand/2	+	+/+	Gor	59
		Bak235/M‡ Δ	27/55	2010	Hand/2	+	−/+	GorΨ	<1
		Bak242/M‡	30/49	2010	Leg/2	+	+/+	Gor	26
		Bak270/M	25/60	2010	Hand/2	+	+/+	Gor	<1
	Bantus	Camvae3/MΔ	25/29	2008	Foot/2	+	−/+	CercoΨ	ND
		Bad316/M‡	36/51	2008	Hand/2	+	+/+	Cpz	ND
		Bad327/M‡ ◊	30/33	2008/10	Multiple/3	+	+/+	Cpz	ND
		Bad332/M‡	25/37	2008	Multiple/3	+	+/+	Gor	31
		Bad348/M‡ ◊	19/27	2008/09	Leg/3	+	+/+	Gor	8
		Bad349/M‡	32/40	2008	Head/2	+	+/+	Gor	41
		Bad350/M◊	40/68	2008/08	Leg/2	+	+/+	Gor	22
		Bad436/M‡	35/56	2009	Hand/2	+	+/+	Cerco	ND
		Bad447/M◊ ‡	40/56	2009/10	Hand/2	+	+/−	Gor	9
		Bad448/M‡ Δ	44/50	2009	Leg/2	+	−/+	GorΨ	<1
		Bad456/M◊ ‡	24/30	2009/10	Leg/2	+	+/+	Gor	23
		Bad463/M‡	37/43	2009	Leg/3	+	+/+	Gor	72
		Bad468.M‡	23/35	2010	Several/3	+	+/+	Gor	26
		Bad551/M	27/38	2010	Arm/2	+	+/+	Gor	<1
General pop	Pygmy	Bobak237/M	−/68	2006	Unknown	+	+/+	Gor	57
	Bantu	Ako254/F‡ ◊	−/65	2008/09	Unknown	+	+/+	Cerco	ND

Pop: population. Loc: location. Cont: contact. Samp: sampling. SFV: Simian Foamy Virus. LTR: Long Terminal Repeat. Gor: Gorilla. Cpz: Chimpanzee. Cerco: *Cercopithecus*/ Ind: Indeterminate/ +: Positive/ −: Negative/ ND: Not determined for Chimpanzees and small monkeys infected individuals. Wound severity: 1 = low, 2 = medium, 3 = high.

◊: sampled twice and tested two times for serology and PCR/.

‡: family members also tested (spouse and/or children).

Δ: LTR was sequenced and blasted. In all cases, correspondence was found with the history.

Φ: (pro)viral loads are reported per 10^5^ cells.

Ψ: No Integrase sequence obtained.

In the “contact group”, the nested PCR on the *pol*-In was positive for 36 samples. The LTR PCR was positive for 37 samples (table 3). Considering both PCRs, SFV DNA (*pol*-In or LTR) was detected in 41 distinct individuals (table 1, table 3). SFV infection prevalence is higher in this group as 18.6% (37/198) individuals were “classically” infected whereas only 0.2% (2/1321) individuals were found to be infected in the “general population” group (X^2^ = 256.7; p = <10^6^). Apes (Gorilla, Chimpanzee) were involved in 37 cases and small monkeys in only 2 cases (Table 3).

The considered classical definition of a SFV infection did not apply to 4 samples in the “contact group” (table 4). Indeed, clear positive results were obtained with both PCRs, but serology was either negative (Bad179, Bad202) or indeterminate (Bad50, Ako394). All four samples were tested in duplicate for the first time and a second sample was collected, one (Bad50, Bad202) and two years (Ako394) later. Individual Bad179 has been missing. Their serological and molecular results profiles were unchanged. Individual Ako394 encountered a gorilla 5 months before the first sampling and reported a scratch on his left thigh. The three other individuals encountered small monkeys during hunting activities. Participant Bad50 was bitten on the arm four years before sampling (table 4).

**Table 4:** Epidemiological and “non-classical” biological features of SFV infected humans in contact group.

Groups	Ethnicity	Samp/sex	Age at: cont/samp	Samp periods	Wound loc/severity	Serology	PCR *pol*-In/LTR	Origin	(Pro)viral LoadΦ
Contact Group	Bantus	Ako394/M* ◊ ‡	53/53	2008/10	Thigh/1	Ind	+/+	Gor	8
		Bad50/M*	56/60	2008/09	Arm/2	Ind	+/+	Cerco	ND
		Bad179/M*	44/46	2008	Leg/2	−	+/+	Cerco	ND
		Bad202/M* ◊	30/31	2008/09	Leg/2	−	+/+	Cerco	ND

Loc: location. Cont: contact. Samp: sampling. SFV: Simian Foamy Virus/ LTR: Long Terminal Repeat/ Gor: Gorilla/ Cerco: *Cercopithecus*/ Ind: Indeterminate/ +: Positive/ −: Negative/ Wound severity: 1 = low, 2 = medium, 3 = high.

*: were serologically tested at least twice with Chimpanzee's antigen. Result profile was invariable. Western Blot with small monkey's (Cercopithecus) antigen was positive for Bad50 and invariably the same for the three others.

◊: sampled twice and tested two times in serology and PCR/.

‡: family members also tested (spouse and/or children).

Φ: (pro)viral loads are reported per 10^5^ cells.

### Viral persistence

For 17 out of the 39 infected individuals in our series, we obtained at least two different samples, after six months (Bak46, Bad350, Bad447, Bad456), after one year (Bak33, Bak56, Bak74, Bak82, Bak132, Bak133, Bobak153, Bak177, Bad348, Ako254), after two years (Bad327) and after three years (Lobak2, Lobak89). In all cases, we found identical serological and molecular positive results. This findings associated to virus isolation in three cases (Réjane Rua Personal data, not shown) demonstrate chronic viral infection in these seventeen individuals harbouring SFV. In all 39 infected persons, viral persistence was estimated to range from 5 months to 45 years (mean = 17 years), based on such findings and the histories collected during field missions.

### (Pro)viral loads

Viral loads were obtained for 33 samples from gorilla infected individuals, using gorilla specific primers. The values, given per 10^5^ cells, ranged from less than 1 to 145 copies, with a mean value of 36 copies/10^5^ cells. No difference in viral load was found according to age at sampling (p = 0.82), ethnicity (p = 0.43), or duration of infection (p = 0.73) (Table 3, 4).

### Foamy-virus sequence analysis and phylogenetic studies

Thirty-eight sequences of the 425 bp fragment of the polymerase gene were obtained from PCR amplicons (GenBank accession numbers JN049028 to JN049065).

Comparative analysis of these new sequences with prototypic strains of African Apes and small monkeys indicates that 30 strains were of gorilla origin (*Gorilla gorilla gorilla*), and 3 from a chimpanzee of the *Pan troglodytes troglodytes* susbspecies, the only type living in these areas. The 5 remaining sequences were from small monkeys, mostly from the *Cercopithecus sp.* A perfect match was found between the histories collected in the “contact group” and the obtained sequences. Only one gorilla sequence was found in individual Bak133 and Bad 468 who were both bitten twice by a different gorilla at different periods. Participant Bad436, who reported a bite by a chimpanzee and one by a small monkey, was found to be infected by a *Cercopithecus* virus. Lastly, two different gorilla sequences (Bak55cl2, Bak55cl3) were amplified from 55 year-old participant Bak55 whose field interviews reported only one remembered gorilla bite but he had been frequently hunting and butchering NHPs throughout his life.

Translation of manually purified sequences resulted in amino acid sequences without stop codons. Pairwise alignments showed a higher percentage of similarity between sequences inside each group and indicated a higher variability among monkey SFVs. Polymorphism analysis among the *Cercopithecus* SFV strains indicated respectively nucleotides and amino acids similarities of 82.1% to 99% and 64.7% to 97.7%. Sequences of the chimpanzees were also all unique and nucleotide similarity ranged from 96% to 99% while it was 91.7% to 99% for amino acids. Concerning the 30 strains of gorilla origin, comparative analysis indicates that while most (25/30) of them differed from each other by some polymorphisms (ranging from 0.3% to 6.4% for nucleotides and 0.8% to 14.3% for amino acids), a few others where identical. Indeed, sequences from Lobak2, Bak46, and Bak56 showed 100% nucleotide identity. Bak46 and Bak56 lived almost 20 km from each other at the time of sampling, while Lobak2 was 130 km and 160 km respectively from the others. Bad348 and Bad349 are two Bantu individuals from the same village who exhibited an identical gorilla sequence. They were bitten both on the same day by the same gorilla. Lastly, a sequence from Bobak153 was identical to a sequence (Cam1083) obtained in an infected hunter, in a previous study performed in a rural village in the low land forest of southern Cameroon [46]. The indicated sampling site (area IX) was around 180 km away from the home of Bobak153, which is also the area where the encounter with the gorilla occurred.

**Figure 5:**
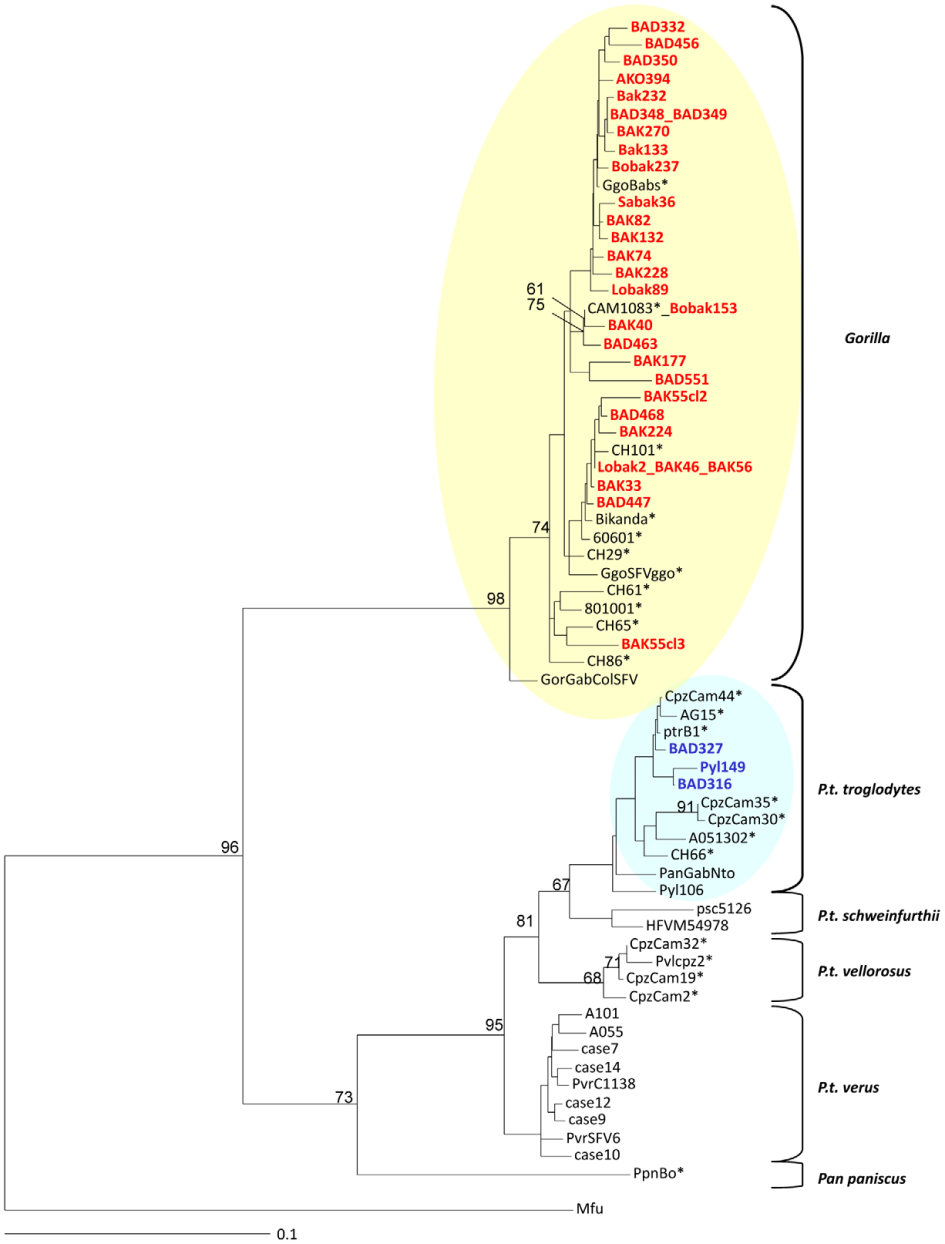
Rooted phylogenetic tree generated with consensus sequences of 425 bp fragments of the SFV *pol*-In from apes-infected hunters and prototype old world non-human primates from the apes' clades. The final consensus sequences from our study are highlighted in red (gorilla) and green (chimpanzee). Sequences are compared to known prototypes from different central African NHP species. Alignment was performed with Dambe version 4.5.68 and Clustal W. Phylogenetic analysis was performed with Paup, version 4.0b10 (Sinauer Associates, Sunderland, MA, USA) based on the Neighbour joining method applying the GTR model. Bootstrap analysis of 1000 replicates is indicated as numbers at nodes. Only values greater than 60% are shown. The scale of the tree is 0,1 nucleotide replacements per site. The tree is rooted on a divergent sequence from an Asian macaque. Species found in Cameroon are shown with asterisks. Sequences accession numbers are: JN049036 to JN049065 for gorilla sequences and JN049033 to JN049035 for Chimpanzee sequences.

**Figure 6:**
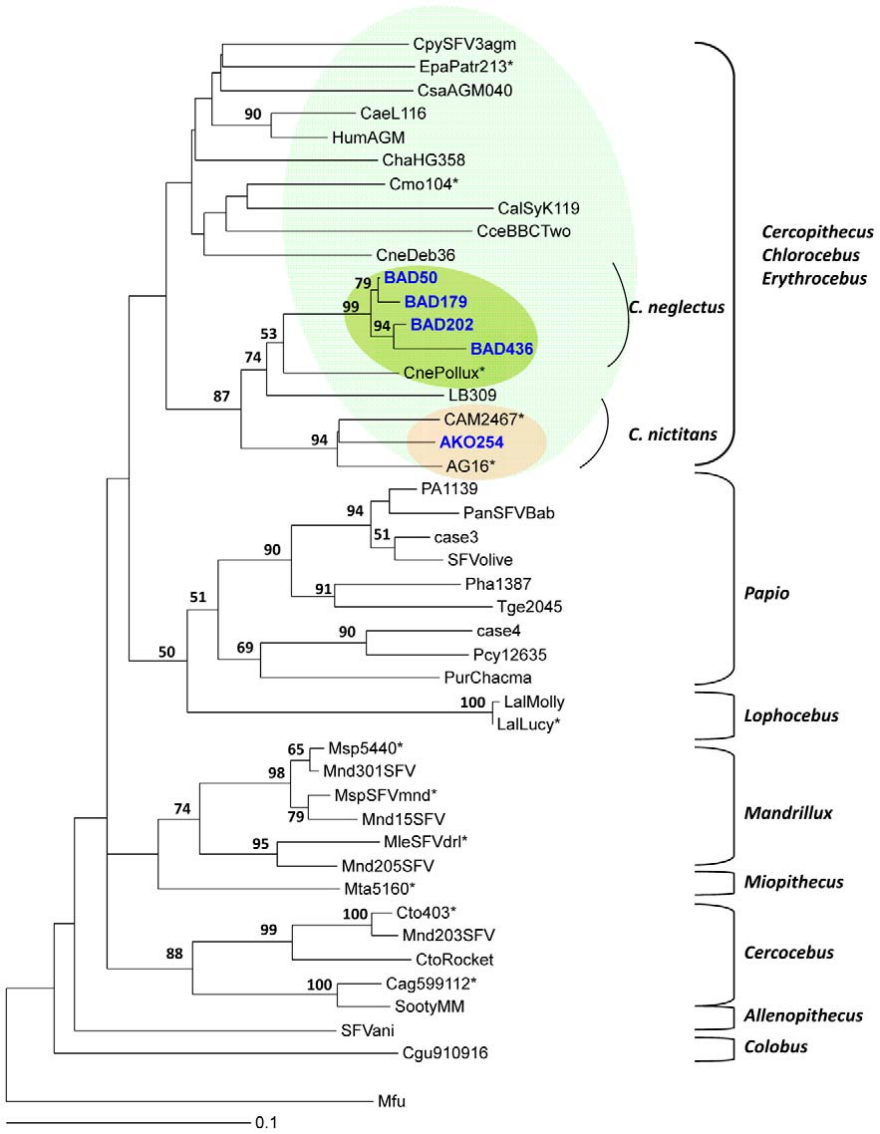
Rooted phylogenetic tree generated with consensus sequences of 425 bp fragments of the SFV *pol*-In from monkeys-infected hunters and prototype old world non-human primates from the small monkeys' clades. The final consensus sequences from our study are highlighted in blue (monkeys). Sequences are compared to known prototypes from different central African NHP species. Alignment was performed with the Dambe version 4.5.68 and Clustal W. Phylogenetic analysis was performed with Paup, version 4.0b10 (Sinauer Associates, Sunderland, MA, USA) based on the Neighbour joining method applying the GTR model. Bootstrap analysis of 1000 replicates is shown as numbers at nodes. Only values greater than 60% are shown. The scale of the tree is 0,1 nucleotides replacement per site. The tree is rooted on a divergent sequence from an Asian macaque. Species found in Cameroon are shown with asterisks. Sequences accession numbers are: JN049028 to JN049032.

Since tree-building algorithms rely on different assumptions, we used two different methods, neighbour-joining (NJ) and maximum likelihood to increase reliability of the derived tree topologies. A first comprehensive study was done with NJ and 1000 bootstrap values, using the 33 new obtained *pol*-In sequences from apes and a selection of prototypic sequences, available in Genbank, from chimpanzees and gorilla. We also included in this study, FV strains from persons infected by African apes. As seen in Figure 5, the different established main clades were identified on the basis of consistent topology and high bootstrap values. Indeed, a first large clade comprised all the strains of gorilla origin, including especially the 30 new ones obtained in our study. A second large clade comprises sequences from all the different chimpanzee subspecies. Clearly, the three chimpanzee strains identified in the current study clustered with the *Pan troglodytes troglodytes* subpecies group strains. A similar tree topology was found using the maximum likelihood method (data not shown). Concerning the small monkey FV sequences, a phylogenetic analysis performed with most of the FVs from African monkeys clearly indicates that the 5 new sequences originated from *Cercopithecus sp*. They cluster into two different subgroups supported by high bootstrap values. A first particular subgroup is supported by a 99% bootstrap value and includes 4 new sequences which represent four newly documented human infections by a *Cercopithecus neglectus* (De Brazza guenon) SFV. The other sequence (Ako254) clusters within the *Cercopithecus nictitans* subgroup, supported by a 94% bootstrap (figure 6).

### Analysis of associated factors

Epidemiological analysis of associated factors for SFV infection in this area was performed on all 1,519 individuals in our series. The univariate analysis of factors associated with SFV infection showed a highly significant association with male sex (p<10^−4^, χ^2^ = 24.6), with the Pygmies (p<10^−4^, χ^2^ = 20.85), hunting (p<10^−4^, χ^2^ = 233.5), apes (p<10^−4^, χ^2^ = 446.2), bites (p<10^−4^, χ^2^ = 267.1) and wounds to the upper part of the body. No significant association was reported for age at contact (table 5). A multivariate model was applied to these combined factors in which only apes (p<10^−4^) and bites (p = 0.005) were independently associated with SFV infection in this series.

**Table 5:** Factors associated to SFV infection in studied populations.

Risk factors	Number of individuals			p
	Tested	Positive	%	
Age at contact				
<50years	783	19	2.43	
>50years	736	18	2.45	0.98
Sex				
Woman	647	1	0.15	
Man	872	36	4.13	<10^−3^
Ethnicity				
Bantus	1084	14	1.29	
Pygmies	412	23	5.29	<10^−3^
Circumstances of contact				
Hunting	190	35	18.42	<10^−5^
Pets	8	0	0	
No contacts	1321	2	0.15	
Type of NHP				
Monkeys	103	2	1.94	<10^−5^
Apes	95	33	34.74	
No contacts	1321	2	0.15	
Type of contact				
Bites	187	31	16.5	<10^−5^
Scratches	6	1	16.6	
Both	5	3	60	
No contacts	1321	2	0.15	
Localisation of the Wound*				
Upper body	114	21	18.3	
Lower body	68	14	20.59	<10^−3^
No contacts	1332	2	0.15	

Univariate analysis was performed with stata. χ^2^ and fisher exact test were realised with a critical *p* value of 0.05.

*missing data in this category (5).

### Secondary intra-familial transmission

A secondary intra-familial transmission was searched for in 12 children aged 9 to 37 years born after the presumed infecting contact and in 30 wives aged 23 to 65 years and who had lived from 1 to more than 30 years with the index case after the presumed infecting contact with the NHP. All these samples were tested serologically. Among the women, only a 51 year-old woman Bad460 (married to Bad447) was clearly sero-positive, while three others, wives of Bobak153, Bak46 and Bak55, were sero-indeterminate. Among the children, a nine year old male child Bak108 (son of Bak40) was sero-indeterminate. Repeated PCR analyses on samples from these five individuals were negative for the *pol*-In and for the LTR. A second sample was collected 6 months later and still showed similar results.

## Discussion

This study reports the largest series yet published, of humans infected with a simian foamy virus, a retrovirus highly endemic in NHPs. Furthermore, this work provides the first data, to our knowledge, concerning the peripheral blood viral load of such retroviral and zoonotic infection in human, by a quantitative PCR method. We report also the negative search for this viral infection in a large series of spouses and children from infected index cases. Lastly, this study reinforces the findings that such zoonotic infection is mainly but interestingly not exclusively acquired through contacts occurring during bites by NHPs. These observations bring out a greater concern on questions concerning the natural history of SFVs in humans:

### 1) What is the magnitude of such human infection in areas highly endemic for infected NHPs, especially in Central Africa?

Concerning central Africa, the work pioneered by Wolfe et al [46], which was followed by our preliminary study [47] identified 16 persons infected by SFVs, as demonstrated by both serological and molecular means. In the present study, we added a series of 39 persons infected by SFVs of NHP origin. Taken together, these data demonstrate FV infection by a wide diversity of NHPs species, in individuals living in different geographical areas of South Cameroon and originating from different ethnic groups (several Bantu groups and two tribes of Pygmies). As only a small proportion of the inhabitants of this large region has been tested for such viruses, it is, however, possible to estimate conservatively that, at least, several hundreds of adults are infected by SFVs in southern Cameroon [55]. The situation is barely known for other African countries. Indeed, apart from one case of infection in a commercial sex worker (CSW) in the Democratic Republic of Congo (DRC)/ex Zaire) [56], only two recent preliminary reports from ongoing studies, indicate the presence of similar zoonotic infection in Gabon (Mouinga-Ondeme, 2011, Abstract Retrovirology) and the DRC (Switzer, 2011, Abstract Retrovirology). Interestingly, in Central Africa, the number of contacts between humans (mostly hunters and their wives and butchers) and NHPs has very probably greatly increased during the last decades [57]. This is mainly due to increased hunting activities, which results from a combination of urban demand for bush-meat, greater access to NHP habitats provided in part by logging roads, easier accessibility to fire arms, and finally, an increase in populations living in forest areas, and the associated increase in local food needs [58]. The results of our study fully support the role played by such factors. Indeed, most (83%) of the SFV infected individuals were relatively young hunters (up to 40 years old) when the presumed infecting contact occurred, but more surprisingly, 16.7% of these contacts occurred within the last 20 years (1991 to 2011). This clearly indicates that hunting NHPs is still an ongoing activity in villages and settlements of southern Cameroon, especially around areas rich in game, such as nature reserves (figure 1). Such hunting activities represent a high-risk occupation for a wide diversity of retroviral zoonotic infections including not only SFVs, but also other retroviral infections such as SIV [16], [17], [59], [60] and STLV [11], [12], [61]. Indeed, even if most of the 16 cumulative cases of human SFV infection reported previously from Cameroon [46], [47] and most of our current 39 cases, were infected by a FV from gorilla (69%, 38/55), at least seven other species of NHPs can also lead to a SFV zoonotic infection in humans. These include chimpanzee, mandrill, baboon and also the most frequently hunted game, including *cercopithecus nictitans*, *cercopithecus cephus*, *cercopithecus neglectus* and some *colobus* and *cercocebus*.

Another point concerning the estimation of the prevalence of SFV infection in human relates to the quite frequent finding of a positive WB serology associated with a negative detection of SFV (in the blood cells) by PCR. Such was the case for 32% (17/53) of WB positive individuals in the “contact group” and 80.7% (21/26) of WB positive persons in the “general population” (data not shown). Whether these persons are infected or not remains unclear. They were not considered as infected in the present study. Similar findings have already been reported and discussed in the literature [46], [47] and might be related to low viral loads in the blood or less likely, to the presence of divergent SFV strains, not recognized by the generic primers used. These primers can detect and amplify a large variety of African SFVs, and also Asian macaque strains [26], [29], [32], [54]. Non-specific reactivity with the SFV Gag proteins (or Gag-only responses) can also be considered as a cause for these profiles.

In this work, we have reported 2 individuals being either sero-indeterminate (only one band by WB) or even sero-negative (2 cases) but in whom we confirmed presence of SFV DNA in their leucocytes blood. Such findings may be related to several factors including a possible long delay of sero-conversion in some cases, especially the individual having been bitten only few months before the sampling. Another possibility could be an individual lack of sero-reactivity for certain proteins as it has been well described in several HTLV-1 or STLV-1 infection, especially for the p24 or some env proteins or peptide [62], [63]. Lastly, in the few persons infected by a *Cercopithecus* monkey foamy strain, this could be linked to the fact that we have used in our WB chimpanzee viral antigens. However, such antigens cross-react strongly to most of the African and Asian SFV yet tested as demonstrated in several published studies [29], [32], [46], [47].

### 2) Is SFV infection pathogenic in humans?

The potential for an SFV infection to cause disease in humans is not yet fully understood. The apparent lack of pathogenicity in infected persons, which is still based on a very limited number of cases [45], [64], contrasts strongly with the massive *in-vitro* lytic properties of these FVs in monkey and human cells [65]. Furthermore, the selection bias inherent in the enrolment of healthy persons in our study, as well as in all of the few published investigations greatly limits the ability to identity any severe acute or chronic diseases. A current case control, based on the series of infected persons reported here, is ongoing to try to detect any potential clinical chronic disease and/or biological abnormalities in persons chronically infected with SFV. However, we have also to keep in mind that the incidence of a disease in a person chronically infected by a retrovirus might be very low and may follow a very long latency. Such features are well exemplified by HTLV-1 infection, another human primate retrovirus of zoonotic origin [66]. Another important issue concerns the possible co-infection by SFV and HIV in the same individual. This has been reported by Switzer et al., in two persons (one CSW and one blood donor) from DRC and Cameroon respectively [56]. Due to the HIV pandemic, in areas where SFV infected persons live (Central Africa and South-East Asia), such co-infections are surely greatly underestimated. Whether HIV-induced immunosuppression could increase the likelihood of developing a disease due to SFV infection remains unknown [55], [56], [67]. Interestingly, cellular tropism of SFV was shown to be enhanced in SIV-induced immunosuppression in a macaque model [37].

### 3) How are SFVs transmitted from apes and monkeys to humans?

As seen above, most (37/39 = 95%) of the persons infected by a SFV had been bitten, often severely, with persisting scars, by a NHP. These data are consistent with our preliminary study in another area of Cameroon, with a severe bite reported in 12/13 infected persons [47]. Similarly, 6 of 8 SFV infected persons in Southeast-Asia reported having been bitten by a macaque, at least once [48]. Furthermore, in persons occupationally exposed to NHPs in primate centres, zoos and laboratories in Germany and North America, the majority of the infected individuals reported also a bite from a NHP [41], [44], [68]. This situation is also exemplified by a recently published study performed in the CIRMF in Gabon [31]. The high rate of infection by gorilla and chimpanzee FVs in our study (17%, 34/198) as compared to other monkeys (1.5%, 3/198), may be related to the severity of the wounds during apes bites. Indeed, in such cases, tissue damage is much more serious (with soft tissue crushing, tearing and bleeding) with possibly deeper and longer contact between apes saliva and blood of the human hunter. In infected monkeys, especially in macaques, studies have provided evidence that SFV is present at high concentrations in saliva and oral mucosa, with viral replication [36], [37], [38]. Furthermore, it appears that in a semi-free colony of macaques, SFV is mostly acquired through severe bites usually in young adults when they compete for sexual partners [32]. A paper also strongly suggests that chimpanzees acquire SFV by horizontal routes, most likely by exposure to saliva [69]. Therefore, all these data indicate that blood and/or injured tissue contact with saliva are the key factor for this form of zoonotic transmission.

No infection was found in the 8 individuals bitten by pets in our study despite 2 WB indeterminate results (data not shown). Pets are usually small sized orphan monkeys, captured at young age, free of infection and brought in the villages where they are raised, away from contacts with infected adult monkeys. Moreover, bites when they happen are almost always superficial.

However, grooming as shown in SFV-infected felines, and possibly to a lesser extent sexual contacts, may also lead to transmission between NHPs [23]. It is thus noteworthy that in our study, as in most of the few other studies reporting SFV infection in humans, some of the infected individuals had not reported severe injuries or bite from a NHP [44], [45], [47], [48]. Furthermore, in some cases, the species that inflicted the injury was not the same as that associated with the infecting SFV strain [45]. Indeed, to our knowledge, a dozen of cases have been reported world-wide among the currently known 85 SFV infected persons, including the 39 from our study. This may suggest that, in some cases, infection occurs through other routes than bites, including from saliva spraying into small open wounds or unprotected muco-cutaneous areas without clear injury. Such a possibility can therefore not be ruled out in the only SFV infected case associated with gorilla scratches in our study (Ako394 table 4). Considering that a majority of people living in West Central Africa frequently butcher, cut or manipulate NHP carcasses or meat [57], [58], other modes of contamination involving external mucosal contacts with infected saliva may be considered. This mode of transmission is likely in this study for the two infected cases in the “general population” series who did not report a NHP bite during their lifetime (Bobak237, Ako254 in table 3). Some transmission routes may be similar to that of transmission for simian herpes B [70]. A better knowledge of such risk factors is important to establish proper protective equipment that should be recommended for worker safety in zoos and primate centres.

### 4) Are these simian viruses transmissible from human to human?

Person to person transmissibility of zoonotic SFV infection remains unclear at present. A major concern of our study was the search for secondary intra-familial transmission from index cases to their close relatives. Indeed, to our knowledge, only 11 spouses of SFV infected persons (6 from workers in North America [45] and 5 in our previous study in Cameroon [47]) and very few of their children that have been tested. They were all found negative for SFV infection.

In our current series, one woman was interestingly found repeatedly (two times at 6 months interval) to be SFV seropositive among the 30 tested wives of SFV infected hunters. This 51 year-old woman had lived for 6 years with index case Bad447, after probable infectious contact and they had not had children. In the absence of a positive PCR with a sequenced SFV DNA fragment comparable to that of her husband, we can not formally rule out, a serological reaction secondary to circumstantial exposition to a virus from the husband. This probably coincidental sero-reactivity could be, as said before, either a non specific Gag reactivity, or a low viral load SFV infection acquired through another route. Besides, the infection may probably not be transmitted given the quite low (pro)viral load (9 copies for 10^5^ cells) observed in the husband (Bad447) (Table 3).

These data indicate that SFV transmission from man to woman does not occur easily by sexual contact or saliva exposure, as previously suggested in the literature [41], [44], [45], [47].

Disease occurrence and transmissibility are related to *in-vivo* (pro)viral load levels in an infected person. We provide here, to our knowledge, the first data concerning the level of (pro)viral load, as determined by a quantitative PCR method, in persons chronically infected by SFV of zoonotic origin. Our results, based on a series of 28 individuals, all infected by a gorilla virus, indicate a low viral load (in the DNA of the peripheral blood cells) of most persons, but with a quite large range (<1 to 145 copies per 10^5^ cells). The degree of this viral load may be related to the origin of the virus (apes versus small monkeys for example) but also to genetic factors, including innate restriction, as already shown for other human retroviral infection of zoonotic origin such as HIV-1 and HTLV-1 [71], [72], [73].

In our series, we have found a women (Ako254) infected by a SFV from a *Cercopithecus*. This is, to our knowledge, the fifth reported case of an infection by a SFV in a woman (as demonstrated by both serological and molecular means) [44], [46], [48]. Moreover, two other reported cases of women living in Southeast Asia and sero-positive for macaque SFV have been reported, without demonstration of viral DNA presence in their blood [48]. All together, these data indicate, as already suggested [44], that SFV might also be spread from mother to child and/or through sexual contacts with infected women.

The demonstration of highly frequent and recent SFV infections by this study raises important public health concerns not only about the risk for the acquisition of SFV, but also about the consequences of such a zoonosis with regard to other simian viruses that may cause disease in humans [4], [58], [67], [74]. This emphasizes the need for continued long term monitoring of SFV infected individuals to evaluate any changes in host and viral dynamics. Although evidence of a secondary transmission are still sought, vigilance must be maintained on the possible emergence of human-to-human transmission from infected individuals, since SFV transmission by blood transfusion has been demonstrated in a monkey model [42], [75] demonstrating infected blood as a mode of virus transmission. Dual infections with SFV and HIV-1 have been reported [56], and the outcome of such an infection in an immunosuppressed person is unknown. Strategies for proactive preparedness for SFV strains that may have the potential for human transmission and clinical outcome must be implemented. Efforts to reduce the risk of cross-species infection are necessary to control the potential threat of new simian pathogens, such as SFVs. Therefore, general public education would be necessary in these areas where interaction with NHP, mostly through hunting is part of culture and tradition, as well as related to economic needs. Preventive actions must then be taken, considering supply alternatives to hunting.
